# Relationship between salivary/pancreatic amylase and body mass index: a systems biology approach

**DOI:** 10.1186/s12916-017-0784-x

**Published:** 2017-02-23

**Authors:** Amélie Bonnefond, Loïc Yengo, Aurélie Dechaume, Mickaël Canouil, Maxime Castelain, Estelle Roger, Frédéric Allegaert, Robert Caiazzo, Violeta Raverdy, Marie Pigeyre, Abdelilah Arredouani, Jean-Michel Borys, Claire Lévy-Marchal, Jacques Weill, Ronan Roussel, Beverley Balkau, Michel Marre, François Pattou, Thierry Brousseau, Philippe Froguel

**Affiliations:** 1 University of Lille, CNRS, Institut Pasteur de Lille, UMR 8199 – EGID, Lille, 59000 France; 2 Institute for Molecular Bioscience, The University of Queensland, Brisbane, 4067 Australia; 3 University of Lille, Inserm, U1190 – EGID, Lille, 59000 France; 4 Endocrine Surgery Department, CHU of Lille, Lille, 59000 France; 5 Qatar Biomedical Research Institute, Qatar Foundation, Doha, 5825 Qatar; 6 Fleurbaix-Laventie Association, Laventie, 62840 France; 7 Inserm CIE 05 – Department of Clinical Epidemiology, Robert Debré Hospital, Paris, 75019 France; 8 Pediatric Endocrine Department, CHU of Lille, Lille, 59000 France; 9 Inserm, U1138, Centre de Recherche des Cordeliers, Paris, 75006 France; 10 Paris-Diderot University, Sorbonne Paris-Cité, Paris, 75013 France; 11 Department of Endocrinology-Diabetology and Nutrition, DHU-FIRE, Bichat Hospital, Assistance Publique-Hôpitaux de Paris, Paris, 75018 France; 12 Inserm, U1018, CESP, Team 5 (EpReC, Renal and cardiovascular Epidemiology), UVSQ-UPS, Villejuif, 94807 France; 13 UF8832 - Biochimie Automatisée, Pôle de Biologie Pathologie Génétique, CHU of Lille, Lille, 59000 France; 14 Department of Genomics of Common Disease, School of Public Health, Imperial College London, Hammersmith Hospital, London, W12 0NN UK

**Keywords:** AMY1A/AMY2A, Body mass index, Copy number variant, Mendelian randomization, Metabonomics, Obesity, Salivary/Pancreatic amylase, Starch

## Abstract

**Background:**

Salivary (AMY1) and pancreatic (AMY2) amylases hydrolyze starch. Copy number of *AMY1A* (encoding AMY1) was reported to be higher in populations with a high-starch diet and reduced in obese people. These results based on quantitative PCR have been challenged recently. We aimed to re-assess the relationship between amylase and adiposity using a systems biology approach.

**Methods:**

We assessed the association between plasma enzymatic activity of AMY1 or AMY2, and several metabolic traits in almost 4000 French individuals from D.E.S.I.R. longitudinal study. The effect of the number of copies of *AMY1A* (encoding AMY1) or *AMY2A* (encoding AMY2) measured through droplet digital PCR was then analyzed on the same parameters in the same study. A Mendelian randomization analysis was also performed. We subsequently assessed the association between *AMY1A* copy number and obesity risk in two case-control studies (5000 samples in total). Finally, we assessed the association between body mass index (BMI)-related plasma metabolites and AMY1 or AMY2 activity.

**Results:**

We evidenced strong associations between AMY1 or AMY2 activity and lower BMI. However, we found a modest contribution of *AMY1A* copy number to lower BMI. Mendelian randomization identified a causal negative effect of BMI on AMY1 and AMY2 activities. Yet, we also found a significant negative contribution of AMY1 activity at baseline to the change in BMI during the 9-year follow-up, and a significant contribution of *AMY1A* copy number to lower obesity risk in children, suggesting a bidirectional relationship between AMY1 activity and adiposity. Metabonomics identified a BMI-independent association between AMY1 activity and lactate, a product of complex carbohydrate fermentation.

**Conclusions:**

These findings provide new insights into the involvement of amylase in adiposity and starch metabolism.

**Electronic supplementary material:**

The online version of this article (doi:10.1186/s12916-017-0784-x) contains supplementary material, which is available to authorized users.

## Background

Amylase is responsible for starch hydrolysis, initiating carbohydrate digestion in the oral cavity and later in the gut. In humans, approximately half of the amylase activity found in serum is produced by the salivary glands and the remaining part by the exocrine pancreas [1] from different genes located in the same complex chromosome 1 locus.

A well-known multi-allelic copy number variant at salivary amylase gene (*AMY1A*; diploid copy number ranging from one to roughly 20) evolved as an adaptation to dietary habits [2]. Populations with high starch consumption carry larger number of copies than others that have maintained an ancestral pre-agricultural way of life [2]. Previously, we reported that *AMY1A* copy number estimated by quantitative RT-PCR (qPCR) is associated with body mass index (BMI) in North European and South Asian adult populations [3]. It provided a putative genetic link between complex carbohydrate metabolism in the gut and obesity. This association was replicated in early-onset obese females from Finland [4] and in prepubertal boys in Italy [5], and an association with insulin resistance was reported in adult Korean men [6], where *AMY1A* copy number was also estimated by qPCR. On the other hand, using digital PCR, two studies failed to reproduce these findings [7, 8]. Usher et al. [7] suggested that the discrepancy with the previously reported observations likely comes from their higher-resolution approaches for both molecular and computational analyses. Recently, however, using digital PCR, we have found that, in Mexican children with high-starch diet, high number of *AMY1A* copies significantly protects against obesity in this population [9]. Finally, a study that used fiber-FISH suggested a role for copy number of pancreatic amylase genes (*AMY2A* and *AMY2B*) in the observed functional associations [10].

This debate is important for several reasons. First, chromosome structural variants are increasingly recognized to highly contribute to disease development [11], and thus the correct genotyping of multi-allelic copy number variant is mandatory [12]. Second, it was shown that non-obese adults with high salivary amylase activity (and putatively high *AMY1A* copy number) present with improved glucose tolerance following liquid starch ingestion [13]. Furthermore, high serum amylase activity was shown to be associated with decreased risk of metabolic syndrome and type 2 diabetes in a Japanese asymptomatic population [14]. Finally, in more than 100 different strains of mice fed a high-fat, high-sucrose diet, the *Amy1* locus was reported to be significantly associated with weight gain variation and with an enrichment of obesity-associated bacteria of gut microbiota [15]. Therefore, it is crucial to robustly determine if amylase activities (and amylase gene copy number) impact energy and glucose homeostasis.

In the present study, we employed a systems biology approach, using genetics, protein activity and metabonomics analyses, to decipher the putative interaction between amylase genes and adiposity in human population. We first assessed the association between plasma enzymatic activity of salivary (AMY1) or pancreatic (AMY2) amylase, and several metabolic traits, including BMI. We then analyzed the effect of *AMY1A* or *AMY2A* copy number on the same parameters. A Mendelian randomization analysis was subsequently performed to assess causality effects explaining the complex relationship between BMI and AMY1 or AMY2 plasma enzymatic activity, and actually suggested a bidirectional causal negative effect in the relationship between BMI and AMY1 plasma enzymatic activity. We subsequently confirmed an association between *AMY1A* copy number and reduced obesity risk in children. Finally, we assessed the association between BMI-related plasma metabolites and AMY1 or AMY2 plasma enzymatic activity.

## Methods

### Study participants

#### D.E.S.I.R

D.E.S.I.R. is a 9-year longitudinal study in a French general population, fully described elsewhere [16]. A total of 4834 unrelated individuals who were successfully genotyped through iSelect Metabochip DNA microarrays (Illumina, San Diego, CA, USA) was included in the present study. *AMY1A* copy number and *AMY2A* copy number were successfully genotyped in 3607 and 3657 participants, respectively. At baseline, we had access to AMY1 plasma enzymatic activity for 3744 participants. Among them, we had access to AMY1 plasma enzymatic activity after 9 years of follow-up for 679 individuals, to BMI after 9 years of follow-up for 2796 individuals, and to the levels of BMI-associated plasma metabolites at baseline for 718 individuals. Moreover, we had access to AMY2 plasma enzymatic activity at baseline for 3980 participants. Among them, we had access to AMY2 plasma enzymatic activity after 9 years of follow-up for 705 individuals, to BMI after 9 years of follow-up for 2970 individuals, and to the levels of BMI-associated plasma metabolites at baseline for 718 individuals. Additional file 1 recapitulates all these numbers. Non-diabetic participants did not use glucose lowering medication, and presented with fasting plasma glucose less than 7 mmol/L and glycated hemoglobin A1c less than 6.5% [17].

#### The Biological Atlas of Severe Obesity study (ABOS)

ABOS is a cohort study (ClinicalGov NCT01129297) from the University Hospital of Lille, France, fully described elsewhere [18]. In the present study, we measured plasma enzymatic activity of AMY1 and AMY2 in 488 participants who were also genotyped through Metabochip DNA microarrays (Illumina).

#### Obesity case-control studies

Clinical characteristics of study participants are shown in Additional file 2. The first case-control study included 2220 normal-weight adults (with a BMI < 25 kg/m^2^) and 1179 adults presenting with obesity (with a BMI ≥ 30 kg/m^2^). These adults were from D.E.S.I.R. or were recruited either by the CNRS UMR8199 (Lille, France), by the Department of Nutrition of Hotel-Dieu Hospital (Paris, France), or by the Centre d'Etude du Polymorphisme Humain (CEPH, Saint-Louis hospital, Paris, France). The second case-control study included 712 normal-weight children or adolescents (with a BMI-for-age < 85th percentile) and 785 children or adolescents presenting with obesity (with a BMI-for-age ≥ 99th percentile). These children or adolescents were from the French Haguenau regional cohort study [19] or from the French Fleurbaix-Laventie Ville Santé study [20], or they were recruited by the CNRS UMR8199 (Lille, France).

### Estimation of AMY1A and AMY2A copy number

Copy number of *AMY1A* and *AMY2A* was estimated using the QX200 droplet digital PCR (ddPCR) system (Bio-Rad Laboratories, Hercules, CA, USA), following the manufacturer’s recommendations. Concentration of DNA samples was measured using the Qubit ds DNA Assay HS kit (Life Technologies, Carlsbad, CA, USA). Dilutions were performed with 20× GE Sample Loading Reagent (Fluidigm, South San Francisco, CA, USA). Each 40 μL reaction included 11 μL ddPCR SuperMix for Probes no dUTP (Bio-Rad), 24 ng DNA (for *AMY1A* copy number estimation) or 32 ng DNA (for *AMY2A* copy number estimation), 1.1 μL of TaqMan assay targeting *AMY1A* or *AMY2A* (Hs07226362_cn or Hs04204136_cn, respectively; Life Technologies), 1.1 μL of TaqMan assay targeting the reference *RNase P* assay (Human *RNase P* #4403328; Life Technologies), and 0.5 U HindIII (High Fidelity; New England Biolabs, Ipswich, MA, USA). Of note, both *AMY1A* and *AMY2A* target assays utilized FAM-labeled probes, while *RNase P* assay was labeled in VIC. Enzymatic digestion was done for 5 minutes at 20 °C. Subsequently, the reaction was emulsified with Droplet Generator Oil (Bio-Rad) using the QX200 Droplet Generator (Bio-Rad), following the manufacturer’s instructions. The droplets were then transferred to a 96-well reaction plate (Eppendorf) and PCR amplification was performed using a Veriti Thermal Cycler (Life Technologies). After amplification, droplets were read using a QX200 Droplet Reader (Bio-Rad). Fluorescence data were analyzed using QuantaSoft software (version 1.7.4, Bio-Rad). Only samples with at least 7000 droplets were kept for further analyses.

### Measurement of plasma enzymatic activities of salivary (AMY1) and pancreatic (AMY2) amylases

Plasma enzymatic activities of total amylase and AMY2 were estimated by an enzymatic colorimetric assay with an autoanalyzer (CoBAS Icobas 8000 modular analyzer series; kits #AMY-P-20766623322 and #AMYL2-03183742122; Hoffman-La Roche, Basel, Switzerland). The plasma enzymatic activity of AMY1 was calculated by subtracting the activity of AMY2 from the activity of total amylase. Normal ranges of the plasma enzymatic activities of AMY2 and total amylase were 13–53 U/L and 29–99 U/L, respectively. Only individuals presenting with these normal ranges were analyzed.

### Measurement of plasma metabolites

Fasting plasma samples were processed by the Metabolon (Durham, NC, USA) platform using gas chromatography mass spectrometry and liquid chromatography-tandem mass spectrometry, as previously described [21, 22]. In the present study, we only analyzed 36 metabolites previously shown to be associated with BMI [23], including 1,5-anhydroglucitol, 1-oleoylglycerophosphocholine (18:1), 2-hydroxybutyrate, 2-linoleoylglycerophosphocholine, 3-(4-hydroxyphenyl)lactate, 3-hydroxyisobutyrate, 3-methyl-2-oxobutyrate, 3-methyl-2-oxovalerate, 4-methyl-2-oxopentanoate, 7-alpha-hydroxy-3-oxo-4-cholestenoate, alpha-hydroxyisovalerate, andro steroid monosulfate 2, asparagine, benzoate, butyrylcarnitine, carnitine, gamma-glutamylisoleucine, gamma-glutamyltyrosine, glutamate, glycerol, glycine, hexanoylcarnitine, histidine, isoleucine, isovalerylcarnitine, kynurenine, lactate, lathosterol, leucine, mannose, N-acetylglycine, palmitoyl sphingomyelin, phenylalanine, propionylcarnitine, tyrosine, and valine. These metabolites presented with a missing value rate of less than 5%. The missing values were imputed with the smallest detected value. We were unable to analyze the BMI-associated metabolite 1-eicosadienoylglycerophosphocholine [23], as it was undetectable in the present study cohort.

### Statistical analyses

#### Ethnic characterization

Ethnic characterization of each participant was assessed using the iSelect Metabochip DNA microarrays (Illumina), as previously described [24].

#### Association analyses between AMY1/AMY2 plasma enzymatic activity or AMY1A/AMY2A copy number and metabolic traits in D.E.S.I.R

The associations between metabolic quantitative traits (except BMI) and enzymatic activity of AMY1/AMY2 or *AMY1A*/*AMY2A* copy number were assessed through linear regression models adjusted for age, sex, BMI, daily alcohol consumption, smoking status, and the first two principal components for ethnicity as previously described [24]. We used the same models for the analysis of plasma metabolites, with the same adjustments (including or not BMI). The analysis of BMI was adjusted for age, sex, daily alcohol consumption, smoking status, and the first two principal components for ethnicity.

The effect of AMY1 or AMY2 activity at baseline on the change in BMI during the 9-year follow-up was assessed through a linear regression model adjusted for age at baseline, sex, BMI at baseline, daily alcohol consumption, smoking status, and the first two principal components for ethnicity. The effect of BMI at baseline on the change in AMY1 or AMY2 activity during the 9-year follow-up was assessed through a linear regression model adjusted for age at baseline, sex, AMY1 or AMY2 activity at baseline, daily alcohol consumption, smoking status, and the first two principal components for ethnicity.

Of note, BMI, aspartate aminotransferase, fasting insulin, triglyceride levels, and the homeostasis model assessment of beta-cell function (HOMA-2B) and of insulin resistance (HOMA-2IR) were logarithmically transformed before statistical analysis.

Association analyses of glucose-related traits were performed in non-diabetic individuals only. Association analyses of lipid traits were performed in participants who did not use any lipid-lowering drugs at baseline. Association analyses of blood pressure were performed in participants who did not use any drugs against hypertension at baseline.

HOMA-2B and HOMA-2IR were calculated in D.E.S.I.R. participants as previously described [24]. In each regression model, traits were analyzed as dependent variables whilst copy number and enzymatic activities were used as covariates.

#### Mendelian randomization analysis between BMI and AMY1/AMY2 plasma enzymatic activity

The causal effect between BMI and AMY1 or AMY2 plasma enzymatic activity was estimated using a Mendelian randomization approach [25, 26].

##### BMI → AMY1/AMY2

We used single nucleotide polymorphisms (SNPs) previously found to be genome-wide significantly associated with BMI [27] as genetic instruments for this analysis. We excluded 14 SNPs with known pleiotropic effects on non-anthropometric traits (Additional file 3). Among the remaining 83 SNPs, four were not testable through the Illumina Metabochip DNA microarray (rs12016871 within *MTIF3* locus, rs16851483 within *RASA2* locus, rs17001654 within *SCARB2* locus, and rs9641123 within *CALCR* locus) and one SNP did not pass the quality control (rs12566985 within the *FPGT* locus). These five SNPs were replaced with proxies (R^2^ ≥ 0.64; Additional file 3). For each of the 83 instrumental genetic variables, we estimated causal effects of BMI on AMY1 or AMY2 plasma enzymatic activity as ratios between the SNP effect sizes on plasma AMY1 or AMY2 enzymatic activity (measured in D.E.S.I.R.) over the SNP effect size on BMI (obtained from Locke et al. [27]). Standard errors for these causal estimates were derived by replacing in the former calculations each SNP effect size on AMY1 or AMY2 plasma enzymatic activity with its corresponding standard error estimated within D.E.S.I.R. The 83 values of causal effects of BMI on AMY1 or AMY2 plasma enzymatic activity were collapsed into single estimates (one for each enzymatic activity) using inverse-variance weighting [25]. Since no published genome-wide association studies on amylase activities were available, we used as an alternative approach, the two-stage least-squares (TSLS) regression to estimate the causal effect of BMI on AMY1 or AMY2 plasma enzymatic activity using D.E.S.I.R. data. This analysis used as the instrumental variable the genetic risk score, calculated as the sum of alleles increasing BMI over the 83 selected SNPs. We did not observe any residual effect of BMI-associated SNPs on amylase activities (*P* > 0.2). To ensure that cryptic pleiotropic effects among the 83 SNPs were not influencing our estimates of causal effect of BMI on AMY1 and AMY2 plasma enzymatic activities, we used Egger regression to test for the significance of the intercept [28]. We found no significant effect of pleiotropy (*P* = 0.41 for AMY1 activity, and *P* = 0.49 for AMY2 activity).

##### AMY1/AMY2 → BMI

We were unable to use *AMY1A* or *AMY2A* copy number as instrumental variables to assess the inverse causation between AMY1 or AMY2 plasma enzymatic activity and BMI as they both showed a residual association with BMI after adjusting for the corresponding plasma enzymatic activity (*P* < 0.001; Additional file 4). We therefore looked for other instruments by testing the association between SNP genotyped on the Metabochip DNA microarray (Illumina) and AMY1 or AMY2 plasma enzymatic activity in the D.E.S.I.R. participants. This association was assessed using linear regression of AMY1 or AMY2 plasma enzymatic activity on genotyped SNP adjusted for age, sex, BMI, and the first two principal components for ethnicity. Subsequently, the significant associations between SNPs and AMY1 or AMY2 plasma enzymatic activity (after Bonferroni correction: *P* < 4 × 10^−7^ = 0.05÷124,571 tested SNPs) were confirmed in ABOS. The combined analyses were performed using a weighted inverse normal method via the function “metagen”, with a fixed effect, in the “META” R package. No heterogeneity was observed (*P* > 0.05). A good instrument was consequently defined as a SNP significantly associated (*P* < 4 × 10^−7^) with AMY1 or AMY2 plasma enzymatic activity, without showing any residual association with BMI (*P* > 0.05). Given these instruments, the causal effect of AMY1 or AMY2 plasma enzymatic activity on BMI was estimated using TSLS regression as implemented in the R package *ivpack* (R function *ivreg*).

#### Association analyses between AMY1A copy number and obesity risk

The association between obesity and *AMY1A* copy number was assessed by a logistic regression model adjusted for age and sex in the two case-control studies. The combined analysis was performed using a weighted inverse normal method via the function “metagen”, with a fixed effect, in the “META” R package. No heterogeneity was observed for this combined analysis (*P* = 0.14).

All genetic analyses were performed under an additive model. All statistical analyses were performed using IBM SPSS (version 14.0) or R (version 3.0).

## Results

### Association study between plasma enzymatic activity of AMY1 or AMY2 and metabolic traits in D.E.S.I.R.

After adjustment for clinical and ethnic confounders, a significant association was found between plasma enzymatic activity of AMY1 or AMY2 and lower BMI in the French D.E.S.I.R. participants (β = –0.0013 ± 0.0002 kg/m^2^, *P* = 2.4 × 10^−13^; β = –0.0024 ± 0.0003 kg/m^2^, *P* = 2.4 × 10^−13^, respectively; Table 1). Importantly, the effects of AMY1 and AMY2 activities seemed partly independent as they remained significant when both AMY1 and AMY2 activities were added in the same regression model (β = –0.0012 ± 0.0002 kg/m^2^, *P* = 2.8 × 10^−12^; β = –0.0021 ± 0.0003 kg/m^2^, *P* = 4.2 × 10^−12^, respectively).

**Table 1:** Association between plasma enzymatic activity of AMY1 or AMY2 and metabolic traits in D.E.S.I.R.

Traits/disorders	AMY1			AMY2		
	n	Effect size ± SE^a^	*P* value	n	Effect size ± SE^a^	*P* value
BMI (kg/m^2^)	3673	−0.0013 ± 0.0002	2.4 × 10^−13^	3905	−0.0024 ± 0.0003	2.0 × 10^−16^
FG (mmol/L)	3629	−0.0019 ± 0.0006	1.4 × 10^−3^	3860	−0.0026 ± 0.0010	9.2 × 10^−3^
FI (pmol/L)	3625	0.0003 ± 0.0006	0.63	3856	0.0025 ± 0.0009	7.5 × 10^−3^
HOMA-2B	3343	0.0010 ± 0.0004	9.0 × 10^−3^	3548	0.0017 ± 0.0006	4.6 × 10^−3^
HOMA-2IR	3343	0.0004 ± 0.0005	0.40	3548	0.0007 ± 0.0008	0.36
HDL (mmol/L)	3432	0.0015 ± 0.0005	2.1 × 10^−3^	3637	−0.0005 ± 0.0008	0.58
LDL (mmol/L)	3427	0.0006 ± 0.0011	0.61	3632	−0.0006 ± 0.0019	0.74
TG (mmol/L)	3463	0.0003 ± 0.0006	0.68	3673	0.0003 ± 0.0010	0.81
ApoB (g/L)	3463	0.0001 ± 0.0003	0.83	3673	−0.0008 ± 0.0005	0.11
ApoA1 (g/L)	3463	0.0008 ± 0.0003	0.016	3673	−0.0009 ± 0.0005	0.10
SBP (mm Hg)	3245	−0.0063 ± 0.017	0.71	3452	−0.067 ± 0.029	0.020
DBP (mm Hg)	3245	0.0010 ± 0.011	0.93	3452	−0.013 ± 0.019	0.48
GGT (IU/L)	3744	−0.0025 ± 0.042	0.95	3980	0.021 ± 0.075	0.79
ALT (IU/L)	3741	0.032 ± 0.021	0.13	3977	−0.025 ± 0.035	0.47
AST (IU/L)	3741	0.0017 ± 0.0004	4.1 × 10^−5^	3977	0.0002 ± 0.0007	0.76

^a^Effect size according to adjusted linear regression model. BMI, AST, FI, HOMA-2B, HO MA-2IR, and TG were logarithmically transformed before statistical analysis

*ALT* alanine aminotransferase, *AMY1* salivary amylase, *AMY2* pancreatic amylase, *ApoA1* apolipoprotein A1, *ApoB* apolipoprotein B, *AST* aspartate aminotransferase, *BMI* body mass index, *CI* confidence interval, *DBP* diastolic blood pressure, *FG* fasting plasma glucose, *FI* fasting serum insulin, *GGT* gamma-glutamyl transferase, *HDL* high-density lipoprotein, *HOMA-2B* homeostasis model assessment of beta-cell function, *HOMA-2IR* homeostasis model assessment of insulin resistance, *LDL* low-density lipoprotein cholesterol, *SBP* systolic blood pressure, *SE* standard error, *TG* triglyceride

Furthermore, in non-diabetic participants, we identified a significant association between AMY1 or AMY2 activity and lower fasting plasma glucose levels (β = –0.0019 ± 0.0006 mmol/L, *P* = 1.4 × 10^−3^; β = –0.0026 ± 0.0010 mmol/L, *P* = 9.2 × 10^−3^, respectively; Table 1), as well as higher beta-cell function modeled by HOMA-2B (β = 0.0010 ± 0.0004, *P* = 9.0 × 10^−3^; β = 0.0017 ± 0.0006, *P* = 4.6 × 10^−3^, respectively; Table 1). These associations remained significant after the inclusion of AMY1 and AMY2 activities in the same regression model (*P* < 0.05). We also found that AMY2 activity was associated with higher fasting serum insulin levels (β = 0.0025 ± 0.0009 pmol/L, *P* = 7.5 × 10^−3^; Table 1). However, neither AMY2 activity nor AMY1 activity were found to be associated with insulin resistance (modeled by HOMA-2IR; *P* > 0.05; Table 1).

Regarding lipid traits, we found that AMY1 activity was associated with higher high-density lipoprotein cholesterol levels (β = 0.0015 ± 0.0005 mmol/L, *P* = 2.1 × 10^−3^; Table 1), as well as higher apolipoprotein A1 levels (β = 0.0008 ± 0.0003 g/L, *P* = 0.016; Table 1).

Furthermore, we identified a significant association between AMY1 activity and higher aspartate aminotransferase levels (β = 0.0017 ± 0.0004 IU/I, *P* = 4.1 × 10^−5^; Table 1), and AMY2 activity was found to be modestly associated with lower systolic blood pressure (SBP; β = -0.067 ± 0.029 mm Hg, *P* = 0.020; Table 1).

### Association study between AMY1A/AMY2A copy number and metabolic traits in D.E.S.I.R.

**Fig. 1:**
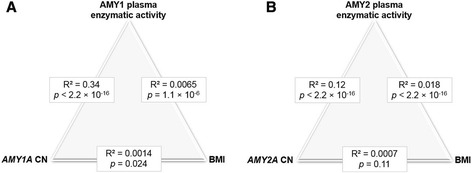
Correlation analyses between (**a**) *AMY1A* copy number, AMY1 activity, and BMI or (**b**) *AMY2A* copy number, AMY2 activity, and BMI in D.E.S.I.R. Data are (unadjusted) Spearman correlations with *P* values

When analyzing copy number of *AMY1A* and *AMY2A* in D.E.S.I.R. participants through ddPCR (Additional files 5 and 6), we confirmed that even *AMY1A* copy numbers were more frequent than odd *AMY1A* copy numbers (Additional files 5 and 6), as shown by Usher et al. [7]. Furthermore, we confirmed that the copy numbers of *AMY1A* and *AMY2A* were nearly always both even or both odd (Additional file 6) [7, 8, 10]. Although *AMY1A* or *AMY2A* copy number was significantly correlated with AMY1 or AMY2 plasma enzymatic activity, respectively (Spearman test: R^2^ = 0.34, *P* < 2.2 × 10^−16^; R^2^ = 0.12, *P* < 2.2 × 10^−16^, respectively; Fig. 1a and b), we only found a nominal association between *AMY1A* copy number and lower BMI (β = –0.0018 ± 0.0009 kg/m^2^ per *AMY1A* copy, *P* = 0.044; Additional file 7). Of note, we found that ddPCR-estimated *AMY1A* copy number is highly correlated with *AMY1A* copy number previously estimated by qPCR (Spearman test: R^2^ = 0.86, *P* < 2.2 × 10^−16^; Additional file 8) in 2137 participants from D.E.S.I.R. [3]. Furthermore, among these 2137 samples previously assessed, we confirmed a highly significant association between ddPCR-estimated *AMY1A* copy number and lower BMI (β = –0.0043 ± 0.0011 kg/m^2^ per *AMY1A* copy, *P* = 1.7 × 10^−4^; Additional files 9 and 10). However, as tackled above, the association between *AMY1A* copy number and lower BMI was only nominal when we analyzed the whole sample set from D.E.S.I.R. (Additional files 7, 9 and 10).

We did not find other significant associations between *AMY1A* or *AMY2A* copy number and metabolic traits (*P* > 0.05; Additional file 7).

### Assessment of the causal effect between AMY1/AMY2 plasma enzymatic activity and BMI in D.E.S.I.R.

A Mendelian randomization analysis was subsequently performed to assess causal effects explaining the complex relationship between BMI and AMY1 or AMY2 activity. We found evidence of a causal negative effect of BMI on both AMY1 (β_causal_ = –4.15 ± 1.66 IU/L per kg/m^2^, *P* = 0.012; Table 2) and AMY2 (β_causal_ = –2.53 ± 0.94 IU/L per kg/m^2^, *P* = 7.1 × 10^−3^; Table 2) plasma enzymatic activities. These significant effects were confirmed using the TSLS regression (Table 2).

**Table 2:** Estimates of causal relationship between BMI and AMY1 or AMY2 plasma enzymatic activity, using two different methods

Relationships to assess	Inverse variance weighting method			Two-stage least squares method		
	Effect size ± SE	*P* value	Instrument	Effect size ± SE	*P* value	Instrument
BMI → AMY1 (n = 3673)	−4.15 ± 1.66	0.012	GRS based on 83 SNPs	−0.97 ± 0.49	0.047	GRS based on 83 SNPs
BMI → AMY2 (n = 3905)	−2.53 ± 0.94	7.1 × 10^−3^	GRS based on 83 SNPs	−0.75 ± 0.28	6.1 × 10^−3^	GRS based on 83 SNPs
AMY1 → BMI (n = 3673)	NA^a^	NA^a^	NA^a^	0.013 ± 0.046	0.78	rs10492100 (*PRH1-PRR4*)
AMY2 → BMI (n = 3905)	NA^a^	NA^a^	NA^a^	0.041 ± 0.097	0.67	rs507666 (*ABO*)

^a^The inverse variance weighting scheme could not be properly applied to estimate the causal effect of AMY1 or AMY2 plasma enzymatic activity on BMI as to our knowledge, no data on association between SNPs and those activities have been published before

*AMY1* salivary amylase, *AMY2* pancreatic amylase, *BMI* body mass index, *GRS* genetic risk score, *NA* not applicable, *SE* standard error, *SNP* single nucleotide polymorphism

Assessing the inverse relationship (AMY1/AMY2 → BMI) turned out to be challenging as we could use neither *AMY1A* nor *AMY2A* copy numbers as genetic instruments. Indeed, we found a significant residual association between *AMY1A* or *AMY2A* copy number and BMI when adjusting for the corresponding plasma enzymatic activity (*P* < 0.001, Additional file 4). As no genome-wide association study for AMY1 or AMY2 plasma enzymatic activity has been performed thus far, we assessed the association between 124,571 SNPs genotyped through the Metabochip DNA microarray and AMY1 or AMY2 activity in D.E.S.I.R. participants, and confirmed the identified associations in another French cohort study (ABOS) in order to find valid genetic instruments. We found one SNP strongly associated with AMY1 activity (*PRH1*-*PRR4* rs10492100: *P* = 3.3 × 10^−11^; Table 3) and two SNPs strongly associated with AMY2 activity (*AMY2B* rs12075225: *P* = 2.0 × 10^−71^; *ABO* rs507666: *P* = 1.3 × 10^−8^; Table 3). SNP rs12075225 could not be considered as instruments into the Mendelian randomization analysis as we found a residual association between rs12075225 and BMI when adjusting for AMY2 activity (*P* < 0.05). However, we were able to use rs10492100 and rs507666 as instruments to assess the causal effect of AMY1 and AMY2 activities, respectively, on BMI. When using TSLS regression with these instruments, we did not find a significant causal relationship of AMY1 or AMY2 activity on BMI (*P* > 0.05; Table 2).

**Table 3:** SNPs found to be significantly associated with AMY1 or AMY2 plasma enzymatic activity in D.E.S.I.R. participants, with a replication study in ABOS participants

	SNP ID	Chr: position (Build 37)	Closest gene	Tested allele/Other allele	Cohort study	n	MAF	Effect size ± SE per allele	*P* value
AMY1	rs10492100	12:11085820	*PRH1-PRR4*	C / T	Discovery	3673	0.21	−2.36 ± 0.39	6.6 × 10^−10^
					Replication	488	0.25	−2.31 ± 0.85	6.9 × 10^−3^
					Meta-analysis	4227	–	−2.35 ± 0.35	3.3 × 10^−11^
AMY2	rs12075225	1:104115017	*AMY2B*	A / C	Discovery	3905	0.10	5.11 ± 0.29	3.3 × 10^−70^
					Replication	488	0.07	3.48 ± 1.04	9.1 × 10^−4^
					Meta-analysis	4227	–	4.99 ± 0.28	2.0 × 10^−71^
AMY2	rs507666	9:136149399	*ABO*	A / G	Discovery	3905	0.22	−1.17 ± 0.22	5.7 × 10^−8^
					Replication	488	0.21	−1.21 ± 0.60	4.4 × 10^−2^
					Meta-analysis	4227	–	−1.17 ± 0.21	1.3 × 10^−8^

*AMY1* salivary amylase, *AMY2* pancreatic amylase, *Chr* chromosome, *MAF* minor allele frequency, *SE* standard error

Next, we took advantage of the prospective D.E.S.I.R. study design with measured AMY1 (n = 679) or AMY2 (n = 705) plasma enzymatic activity after 9 years of follow-up (Additional file 1). Indeed, although confounding can still be present in prospective studies, having consistent results between baseline and follow-up data reinforces the significance of the causal effect estimated besides using Mendelian randomization tools. We found a significant negative effect of BMI at baseline on the change in AMY1 activity (β = –0.20 ± 0.08 IU/L, *P* = 0.014) or AMY2 activity (β = –0.18 ± 0.06 IU/L, *P* = 3.0 × 10^−3^) during the 9-year follow-up, which is in line with the results of the Mendelian randomization analyses showing that BMI negatively impacts amylase activity. Nonetheless, we also identified a significant negative contribution of AMY1 activity at baseline to the change in BMI during the 9-year follow-up (n = 2796; β = –0.0062 ± 0.0027 kg/m^2^, *P* = 0.022), which would imply a bidirectional causal negative effect in the relationship between BMI and AMY1 plasma enzymatic activity. The association between AMY2 activity at baseline and the change in BMI during the 9-year follow-up was not significant (*P* > 0.05).

### Association study between AMY1A copy number and obesity in adults and children/adolescents

The uncertainty about the causal effect of lower *AMY1A* copy number (or AMY1 activity) to higher BMI prompted us to assess the association between *AMY1A* copy number and obesity risk in two French case-control studies, one including 1179 obese adults and 2220 controls, and the other one including 785 obese children/adolescents and 712 controls (Additional file 2; Additional files 11 and 12). In the French adults, we found that the mean number of *AMY1A* copies was lower in obese subjects (6.8 ± 2.5; Table 4) than in controls (7.0 ± 2.6; Table 4), although this difference was not significant when we adjusted the logistic regression model for both age and sex (*P* = 0.13; Table 4). In contrast, in the French children/adolescents, we found a significant association between *AMY1A* copy number and lower obesity risk (odds ratio (OR) per estimated copy 0.94; 95% confidence interval (CI), 0.90–0.98; *P* = 7.1 × 10^−3^; Table 4). When we combined the two case-control studies in adults and youths, we identified a significant contribution of *AMY1A* copy number to lower obesity risk (OR per estimated copy 0.97; 95% CI, 0.94–0.99; *P* = 6.8 × 10^−3^; heterogeneity: *P* = 0.14; Table 4).

**Table 4:** Association between *AMY1A* copy number and obesity risk

Study	Obese cases		Controls		OR (95% CI)^b^	*P* value
	n	*AMY1A* copies^a^	n	*AMY1A* copies^a^		
Adults	1179	6.8 ± 2.5	2220	7.0 ± 2.6	0.98 (0.95–1.01)	0.13
Children/Adolescents	785	7.0 ± 2.6	712	7.3 ± 2.7	0.94 (0.90–0.98)	7.1 × 10^−3^
Combined analysis	–	–	–	–	0.97 (0.94–0.99)	6.8 × 10^−3^

^a^Data are means ± standard deviation

^b^Odds ratio per *AMY1A* copy from a logistic regression adjusted for age and sex

*AMY1A* salivary amylase gene, *CI* confidence interval, *OR* odds ratio

### Association analysis between known BMI-associated plasma metabolites and AMY1/AMY2 plasma enzymatic activity in D.E.S.I.R.

Finally, we aimed to assess the association between 36 plasma metabolites known to be associated with BMI [23] and AMY1 or AMY2 plasma enzymatic activity in 718 D.E.S.I.R. participants. First, we confirmed a significant association between these metabolites and BMI in these participants, except for palmitoyl sphingomyelin, which was found to be only metabolite nominally associated with BMI (*P* = 0.07), although with the same published effect size direction (β < 0) [23]. Then, we identified significant associations between several metabolites, including branched-chain amino acids (isoleucine, isovalerylcarnitine, and leucine), and AMY1 and/or AMY2 plasma enzymatic activity (Additional file 13), with an effect size direction opposite to the one of BMI effect on the same metabolites (Additional file 13). Interestingly, lactate was significantly associated with higher AMY1 activity, when the regression model was adjusted or not for BMI (β = 0.050 ± 0.020, *P* = 5.8 × 10^−3^; BMI-adjusted: β = 0.058 ± 0.020, *P* = 1.6 × 10^−3^; Additional file 13).

## Discussion

In the present study, we found that plasma enzymatic activities of both AMY1 and AMY2 were markedly associated with lower BMI and some other related metabolic traits, including lower fasting plasma glucose levels, higher pancreatic beta-cell function, and better lipid profiles, linking starch hydrolysis and metabolism in humans. Although AMY1 or AMY2 plasma enzymatic activity was significantly correlated with the number of copies of *AMY1A* or *AMY2A*, respectively (Fig. 1a and b), we found a nominal association between *AMY1A* copy number and lower BMI in middle-aged French adults. However, we identified a significant association between *AMY1A* copy number and lower risk of obesity in French children, which is in line with our previous study performed in Mexican children [9].

The present study also assessed the hypothesis that pancreatic amylase genes (instead of salivary amylase gene) could actually drive the association with BMI [10]. However, we did not find any significant marginal association between *AMY2A* copy number and BMI, which makes unlikely a major role for pancreatic amylase gene. Yet, we only genotyped *AMY2A* copy number and not *AMY2B* copy number, which is a limitation of our study, even if Usher et al. [7] showed that *AMY2A* copy number was similar to *AMY2B* copy number in approximately 95% of haploid genotypes.

Through a Mendelian randomization analysis, we identified a causal negative effect of BMI on plasma enzymatic activities of both AMY1 and AMY2. In contrast, we failed to find any causal effect of AMY1 or AMY2 plasma enzymatic activity on BMI, although this specific analysis likely lacked sufficient statistical power. Indeed, since we could use neither *AMY1A* nor *AMY2A* copy number as a genetic instrument for the analysis, we were deprived of the possibility to utilize some of the strongest potential instruments available. Despite this limitation, we were able to find surrogate instruments that were, however, poorly associated with plasma enzymatic activities of AMY1 and AMY2 compared to their corresponding gene copy number. In addition, since no large genome-wide association study for AMY1 or AMY2 plasma enzymatic activity has been performed so far, we were left with a very limited number of useable instruments. The prospective data available in D.E.S.I.R. further supported the negative effect of BMI on AMY1 and AMY2 activities. However, we also found a significant negative contribution of AMY1 activity at baseline to the change in BMI during the 9 years of follow-up, which implies a possibly causative impact of AMY1 activity on decreased adiposity. This was supported by the present results obtained from our obesity case-control study, showing a significant contribution of *AMY1A* copy number to decreased obesity risk in French children.

The impact of AMY1 activity and *AMY1A* copy number on adiposity is therefore complex and it seems to interact with the metabolic effect of complex carbohydrate digestion by the gut microflora. Our recent independent digital PCR analyses of *AMY1A* copy number in Mexican children [9] and our present results in French youths found strong evidence that high *AMY1A* copy numbers protect against childhood obesity in this high-starch diet populations. In contrast, in French middle-aged adults from the general population, we failed to reproduce these findings. The difference between adults and children may be due to the fact that the heritability of BMI is higher in childhood than in adulthood [29, 30], optimizing the identification of significant associations between genetic events and obesity risk. Furthermore, this difference may be due to different gene–environment interactions depending on age [29, 30]. For instance, youths may eat more carbohydrates than adults (as the energy requirements of youths have been shown to parallel their growth rate) [31]. In rodents, it was shown that the SNP at *Amy1* locus strongly predicts weight gain after 8 weeks on a high-fat, high-sucrose diet, with an associated enrichment in gut bacteria observed in obesity states, which may mediate the metabolic effect of *Amy1* expression variation [15].

In D.E.S.I.R., we found that AMY1 plasma enzymatic activity was significantly associated with higher plasma lactate levels independently of BMI. Lactate is a well-known product of complex carbohydrate fermentation by the gut microbiota [32]. It has been proposed that decrease in the lactate/butyrate ratio can generate an extra 20 calories/day, which may lead to an extra kilogram for weight over a year [32]. Therefore, we suggest that amylase activity, which is associated with higher lactate production, may protect against obesity, especially in individuals with a high-starch diet.

## Conclusions

In conclusion, our systems biology study performed in a prospectively followed population-based European cohort suggests a bidirectional relationship between AMY1 plasma enzymatic activity and adiposity. Altogether, low AMY1 activity due to both genetic and environmental events may modulate human colonic microbiota fermentation of oligosaccharides into short-chain fatty acids via lactate regulation [32], which may have a negative impact on energy harvest, and therefore may aggravate obesity. Further studies are warranted to assess the validity of this hypothesis that, if confirmed, may have clinical implications in obesity treatment [33].

## Additional files

Additional file 1: D.E.S.I.R. participants included in the study at baseline and after 9 years of follow-up. (DOC 203 kb)
Additional file 2: Clinical characteristics of participants included in the two obesity case-control studies. (DOC 30 kb)
Additional file 3: Effect of SNPs previously found to be associated with BMI on BMI variation in D.E.S.I.R. (DOC 94 kb)
Additional file 4: Association between copy number of *AMY1A* or *AMY2A* and BMI in D.E.S.I.R., respectively adjusted for plasma enzymatic activity of AMY1 or AMY2. (DOC 28 kb)
Additional file 5: Copy number distributions of (A) *AMY1A* and (B) *AMY2A* in D.E.S.I.R. (DOC 185 kb)
Additional file 6: Unrounded copy number estimates for *AMY1A* and *AMY2A* in D.E.S.I.R. (DOC 206 kb)
Additional file 7: Association between copy number of *AMY1A* or *AMY2A* and metabolic traits in D.E.S.I.R. (DOC 46 kb)
Additional file 8: Correlation between *AMY1A* copy number estimated by qPCR versus ddPCR. (DOC 700 kb)
Additional file 9: Association between ddPCR-estimated *AMY1A* copy number and BMI in several sets of D.E.S.I.R.: (i) the first set of 2137 samples previously analyzed by qPCR in Falchi et al. [3] paper; (ii) the second set of “all samples minus those 2137 samples”, and (iii) all samples from D.E.S.I.R. (DOC 31 kb)
Additional file 10: BMI (means ± s.e.m.) according to ddPCR-estimated *AMY1A* copy number in several sets of D.E.S.I.R.: (i) the first set of 2137 samples previously analyzed by qPCR in Falchi et al. [3] paper; (ii) the second set of “all samples minus those 2137 samples (=1463 samples)”, and (iii) all samples from D.E.S.I.R. (DOC 187 kb)
Additional file 11: Distribution of *AMY1A* copy number in (A) 1179 obese adults and 2220 controls; (B) 785 obese children/adolescents and 712 controls. (DOC 59 kb)
Additional file 12: Unrounded *AMY1A* copy number estimates in (A) 1179 obese adults and 2220 controls; (B) 785 obese children/adolescents and 712 controls. (DOC 154 kb)
Additional file 13: Significant associations between BMI-associated plasma metabolites and AMY1 or AMY2 plasma enzymatic activity in 718 participants from D.E.S.I.R. (DOC 47 kb)

## Abbreviations

AMY1: salivary amylase
AMY1A: salivary amylase gene
AMY2: pancreatic amylase
AMY2A: pancreatic amylase gene
BMI: body mass index
ddPCR: droplet digital PCR
HOMA-2B: homeostasis model assessment of beta-cell function
HOMA-2IR: homeostasis model assessment of insulin resistance
qPCR: quantitative RT-PCR
SNP: single nucleotide polymorphism
TSLS: two-stage least-squares.
