# Supplementary material for: Relationship between salivary/pancreatic amylase and body mass index: a systems biology approach
Source: BMC Med. 2017 Feb 23;15:37. doi: 10.1186/s12916-017-0784-x (PMC5322607; doi:10.1186/s12916-017-0784-x)

**Additional file 1. D.E.S.I.R. participants included in the study at baseline and after nine years of follow-up**


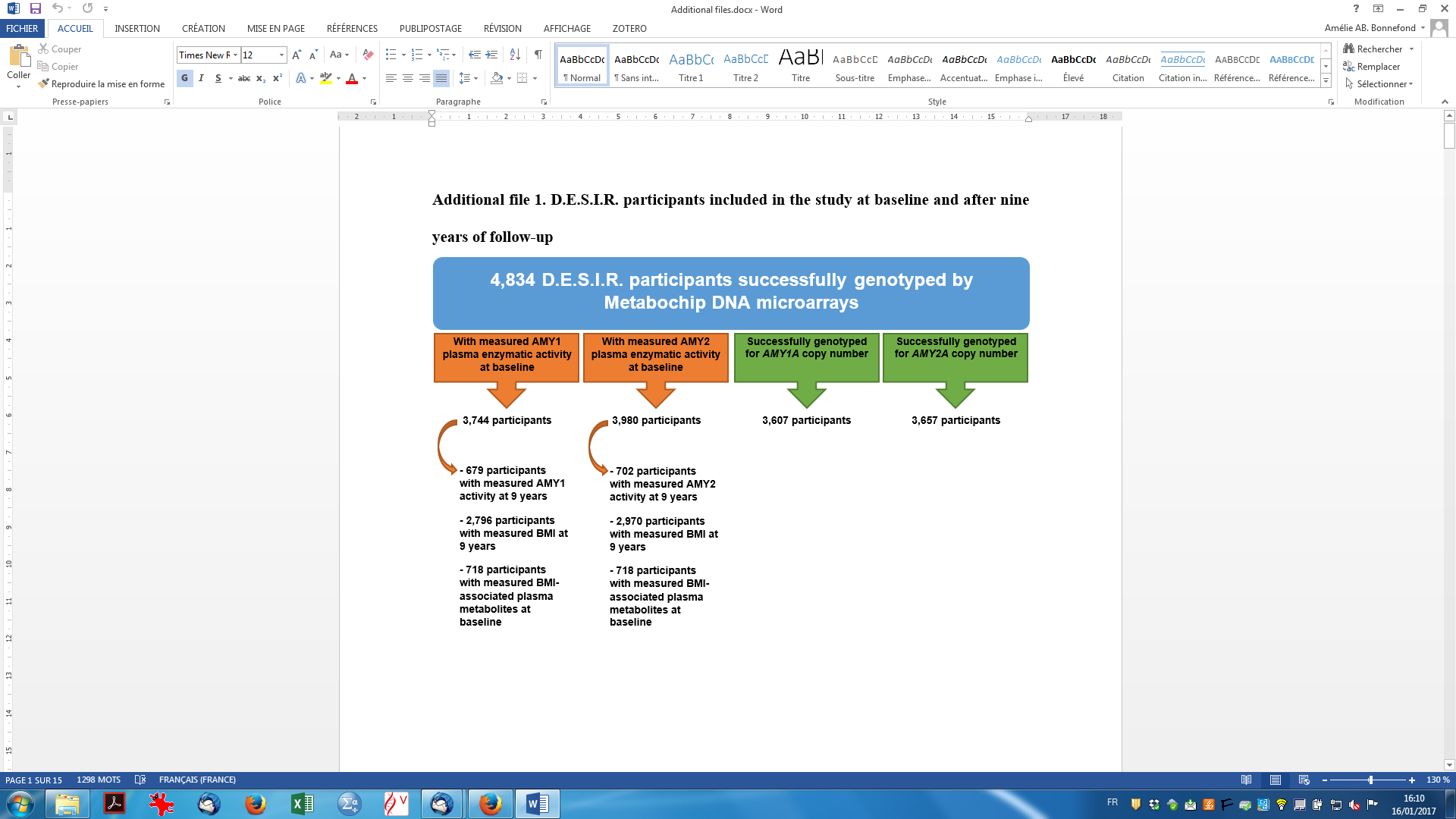

Supplement: Additional file 1: — D.E.S.I.R. participants included in the study at baseline and after 9 years of follow-up. (DOC 203 kb) [file 12916_2017_784_MOESM1_ESM.doc]
