# Supplementary material for: Relationship between salivary/pancreatic amylase and body mass index: a systems biology approach
Source: BMC Med. 2017 Feb 23;15:37. doi: 10.1186/s12916-017-0784-x (PMC5322607; doi:10.1186/s12916-017-0784-x)
Supplement: Additional file 2: — Clinical characteristics of participants included in the two obesity case-control studies. (DOC 30 kb) [file 12916_2017_784_MOESM2_ESM.doc]

**Additional file** 2. Clinical characteristics of participants included in the two obesity case-control studies

| **Study** | **Obese cases** | | | **Controls** | | |
| --- | --- | --- | --- | --- | --- | --- |
|  | ***n***  **(M / F)** | **Age*** | **BMI*** | ***n***  **(M / F)** | **Age*** | **BMI*** |
| Adults | 1,179  (374 / 805) | 50.1 ± 12.3 | 39.0  [32.6 – 45.2] | 2,220  (897 / 1,323) | 45.5 ± 9.9 | 22.5  [21.0 – 23.8] |
| Children/ Adolescents | 785  (356 / 429) | 12.1 ± 2.6 | 30.9  [27.8 – 34.2] | 712  (364 / 348) | 15.5 ± 3.1 | 19.6  [17.9 – 21.2] |

*Data are means ± standard deviation or median [25^th^ percentile – 75^th^ percentile]

***BMI*,** body mass index; ***F***, females; ***M***, males.
