# Supplementary material for: Relationship between salivary/pancreatic amylase and body mass index: a systems biology approach
Source: BMC Med. 2017 Feb 23;15:37. doi: 10.1186/s12916-017-0784-x (PMC5322607; doi:10.1186/s12916-017-0784-x)
Supplement: Additional file 3: — Effect of SNPs previously found to be associated with BMI on BMI variation in D.E.S.I.R. (DOC 94 kb) [file 12916_2017_784_MOESM3_ESM.doc]

**Additional file 3. Effect of SNPs previously found to be associated with BMI on BMI variation in D.E.S.I.R.**

- Association between the GRS including the 83 SNPs (without pleiotropic effect) and BMI in D.E.S.I.R.: β = 0.075 kg/m² per allele; *p* = 1.52E-19 (adjusted for age, sex and ethnicity).
- Association between the GRS including all SNPs (with and without pleiotropic effect) and BMI in D.E.S.I.R.: β = 0.081 kg/m² per allele; *p* = 4.88E-19 (adjusted for age, sex and ethnicity).
