# Supplementary material for: Relationship between salivary/pancreatic amylase and body mass index: a systems biology approach
Source: BMC Med. 2017 Feb 23;15:37. doi: 10.1186/s12916-017-0784-x (PMC5322607; doi:10.1186/s12916-017-0784-x)
Supplement: Additional file 4: — Association between copy number of AMY1A or AMY2A and BMI in D.E.S.I.R., respectively adjusted for plasma enzymatic activity of AMY1 or AMY2. (DOC 28 kb) [file 12916_2017_784_MOESM4_ESM.doc]

**Additional file 4. Association between copy number of *AMY1A* or *AMY2A* and BMI in D.E.S.I.R., respectively adjusted for** plasma enzymatic activity of AMY1 or AMY2

| **Trait** | ***AMY1A*** | | ***AMY2A*** | |
| --- | --- | --- | --- | --- |
|  | **Effect size ± SE***  **per *AMY1A* copy** | ***p*-value** | **Effect size ± SE***  **per *AMY2A* copy** | ***p*-value** |
| BMI (kg/m²) | **0.0048 ± 0.0012** | **6.7 × 10^-5^** | **0.031 ± 0.005** | **4.7 × 10^-9^** |

*Effect size according to linear regression model adjusted for age, sex, daily alcohol consumption, current smoking status, plasma enzymatic activity of AMY1 or AMY2, and the first two principal components for ethnicity;

BMI was logarithmically transformed before statistical analysis.

***AMY1A***, salivary amylase gene; ***AMY2A***, pancreatic amylase gene; ***BMI***, body mass index; ***SE***, standard error.
