# Supplementary material for: Relationship between salivary/pancreatic amylase and body mass index: a systems biology approach
Source: BMC Med. 2017 Feb 23;15:37. doi: 10.1186/s12916-017-0784-x (PMC5322607; doi:10.1186/s12916-017-0784-x)
Supplement: Additional file 5: — Copy number distributions of (A) AMY1A and (B) AMY2A in D.E.S.I.R. (DOC 185 kb) [file 12916_2017_784_MOESM5_ESM.doc]

**Additional file 5. Copy number distributions of (A) *AMY1A* and (B) *AMY2A* in D.E.S.I.R.**


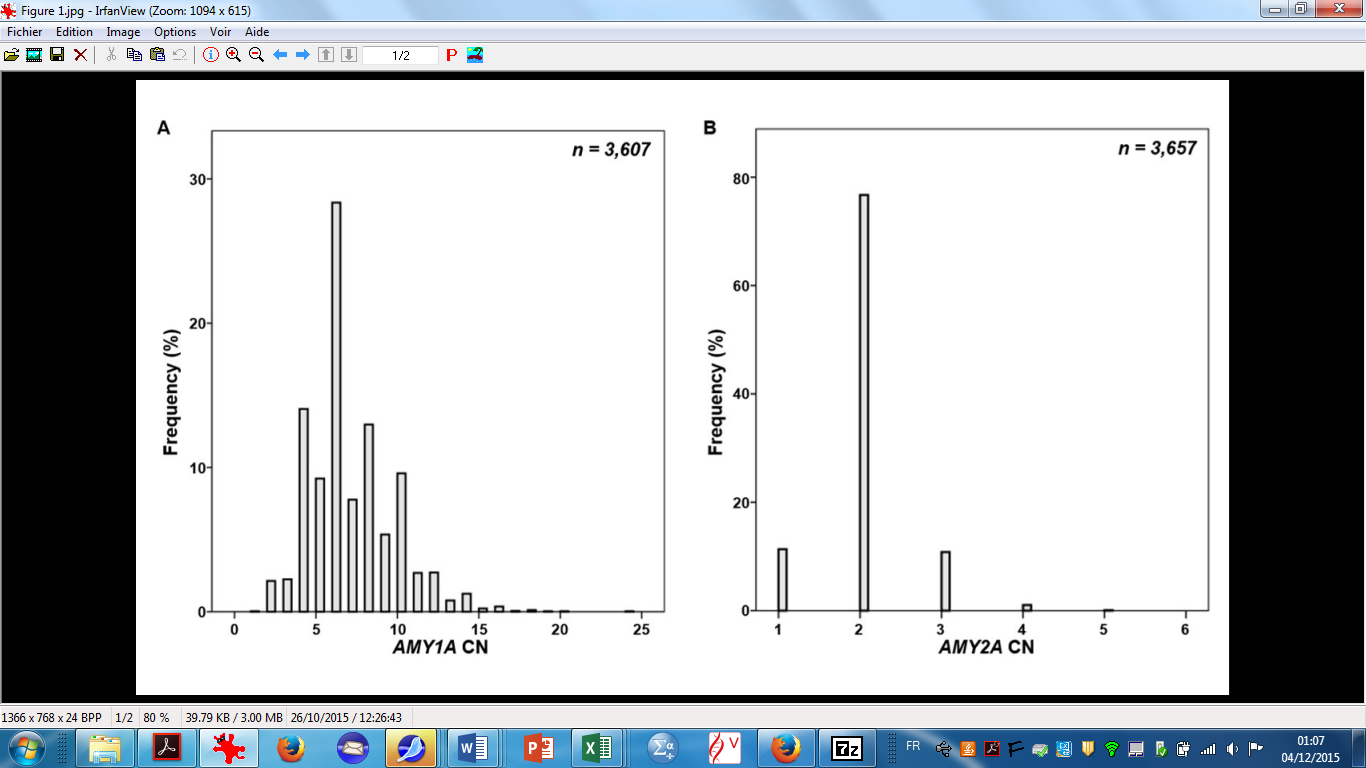


***AMY1A***, salivary amylase gene; ***AMY2A***, pancreatic amylase gene; ***CN***, copy number.
