# Supplementary figures and images for: Relationship between salivary/pancreatic amylase and body mass index: a systems biology approach
Source: BMC Med. 2017 Feb 23;15:37. doi: 10.1186/s12916-017-0784-x (PMC5322607; doi:10.1186/s12916-017-0784-x)

**Additional file 6. Unrounded copy number estimates for *AMY1A* and *AMY2A* in D.E.S.I.R.**


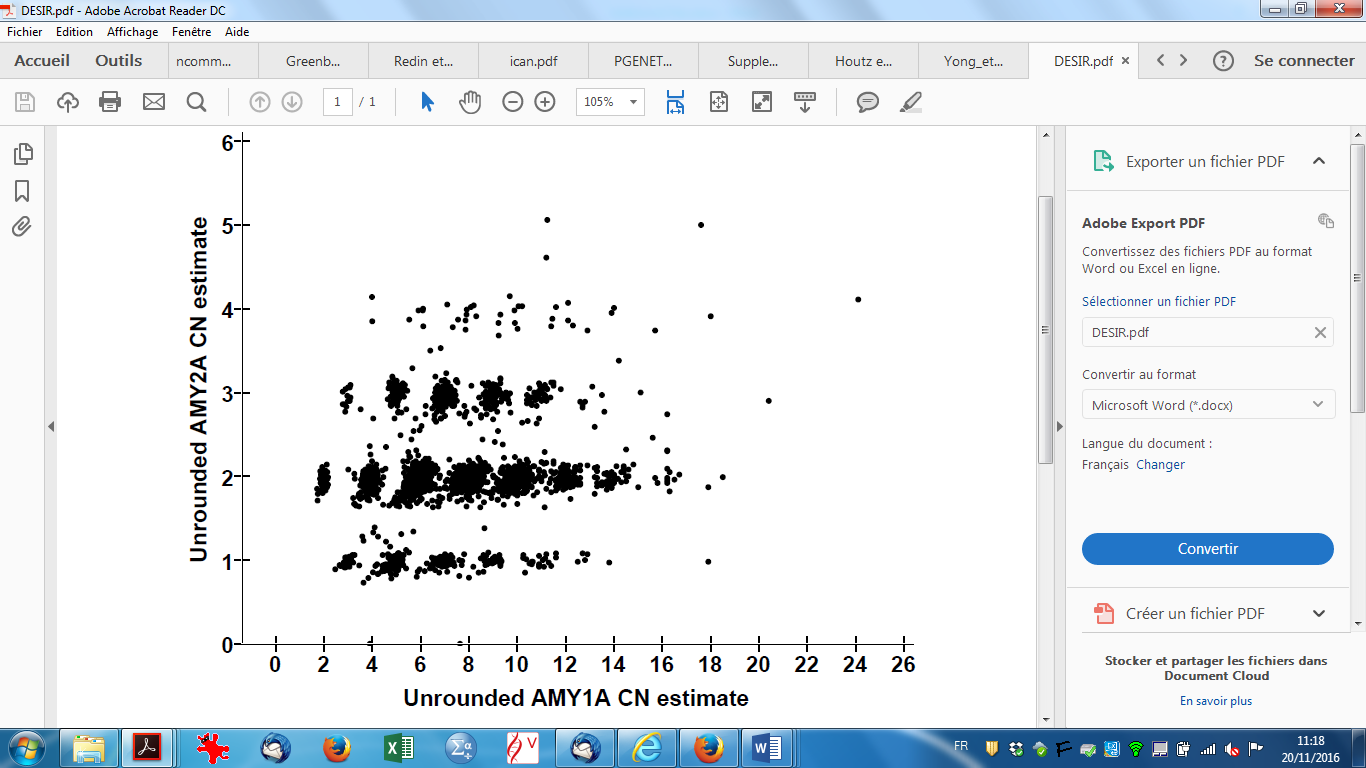


***CN***, copy number.

Supplement: Additional file 6: — Unrounded copy number estimates for AMY1A and AMY2A in D.E.S.I.R. (DOC 206 kb) [file 12916_2017_784_MOESM6_ESM.doc]
