# Supplementary material for: Relationship between salivary/pancreatic amylase and body mass index: a systems biology approach
Source: BMC Med. 2017 Feb 23;15:37. doi: 10.1186/s12916-017-0784-x (PMC5322607; doi:10.1186/s12916-017-0784-x)
Supplement: Additional file 7: — Association between copy number of AMY1A or AMY2A and metabolic traits in D.E.S.I.R. (DOC 46 kb) [file 12916_2017_784_MOESM7_ESM.doc]

**Additional file 7. Association between copy number of *AMY1A* or *AMY2A*** and metabolic traits in D.E.S.I.R.

| **Traits/disorders** | ***AMY1A*** | | | ***AMY2A*** | | |
| --- | --- | --- | --- | --- | --- | --- |
|  | ***n*** | **Effect size ± SE***  **per *AMY1A* copy** | ***p*-value** | ***n*** | **Effect size ± SE***  **per *AMY2A* copy** | ***p*-value** |
| BMI (kg/m²) | 3,600 | **-0.0018 ± 0.0009** | **0.044** | 3,650 | 0.0064 ± 0.0043 | 0.14 |
| FG (mmol/l) | 3,504 | -0.0025 ± 0.0031 | 0.42 | 3,548 | 0.020 ± 0.015 | 0.19 |
| FI (pmol/l) | 3,501 | 0.0018 ± 0.0029 | 0.53 | 3,546 | 0.014 ± 0.015 | 0.35 |
| HOMA-2B | 3,212 | 0.0015 ± 0.0019 | 0.43 | 3,259 | -0.0061 ± 0.0093 | 0.51 |
| HOMA-2IR | 3,212 | 0.0020 ± 0.0026 | 0.45 | 3,259 | 0.0021 ± 0.013 | 0.87 |
| HDL (mmol/l) | 3,289 | 0.0005 ± 0.0026 | 0.84 | 3,339 | 0.011 ± 0.013 | 0.39 |
| LDL (mmol/l) | 3,284 | 0.0046 ± 0.0057 | 0.42 | 3,336 | -0.016 ± 0.028 | 0.57 |
| TG (mmol/l) | 3,315 | 0.0010 ± 0.0032 | 0.75 | 3,365 | 0.0013 ± 0.016 | 0.93 |
| ApoB (g/l) | 3,314 | 0.0007 ± 0.0016 | 0.68 | 3,364 | -0.0032 ± 0.0081 | 0.69 |
| ApoA1 (g/l) | 3,314 | 0.0020 ± 0.0016 | 0.22 | 3,364 | 0.0065 ± 0.0081 | 0.42 |
| SBP (mm Hg) | 3,209 | 0.018 ± 0.086 | 0.83 | 3,248 | -0.71 ± 0.43 | 0.10 |
| DBP (mm Hg) | 3,209 | 0.036 ± 0.056 | 0.52 | 3,248 | -0.23 ± 0.28 | 0.41 |
| GGT (IU/I) | 3,607 | -0.18 ± 0.21 | 0.39 | 3,657 | 0.55 ± 1.09 | 0.62 |
| ALT (IU/I) | 3,606 | -0.035 ± 0.095 | 0.71 | 3,656 | -0.54 ± 0.49 | 0.26 |
| AST (IU/I) | 3,606 | -0.0006 ± 0.0021 | 0.78 | 3,656 | -0.011 ± 0.011 | 0.29 |

*Effect size according to adjusted linear regression model. BMI, AST, FI, HOMA-2B, HOMA-2IR and TG were logarithmically transformed before statistical analysis.

***AMY1A***, salivary amylase gene; ***AMY2A***, pancreatic amylase gene; ***ALT***, alanine aminotransferase; ***ApoA1***, apolipoprotein A1; ***ApoB***, apolipoprotein B; ***AST***, aspartate aminotransferase; ***BMI***, body mass index; ***CI***, confidence interval; ***DBP***, diastolic blood pressure; ***FG***, fasting plasma glucose; ***FI***, fasting serum insulin; ***GGT***, gamma-glutamyl transferase; ***HDL***, high-density lipoprotein; ***HOMA-2B***, homeostasis model assessment of beta-cell function; ***HOMA-2IR***, homeostasis model assessment of insulin resistance; ***LDL***, low-density lipoprotein cholesterol; ***SBP***, systolic blood pressure; ***SE***, standard error; ***TG***, triglyceride.
