# Supplementary material for: Relationship between salivary/pancreatic amylase and body mass index: a systems biology approach
Source: BMC Med. 2017 Feb 23;15:37. doi: 10.1186/s12916-017-0784-x (PMC5322607; doi:10.1186/s12916-017-0784-x)
Supplement: Additional file 8: — Correlation between AMY1A copy number estimated by qPCR versus ddPCR. (DOC 700 kb) [file 12916_2017_784_MOESM8_ESM.doc]

**Additional file 8. Correlation between *AMY1A* copy number estimated by qPCR *versus* ddPCR**


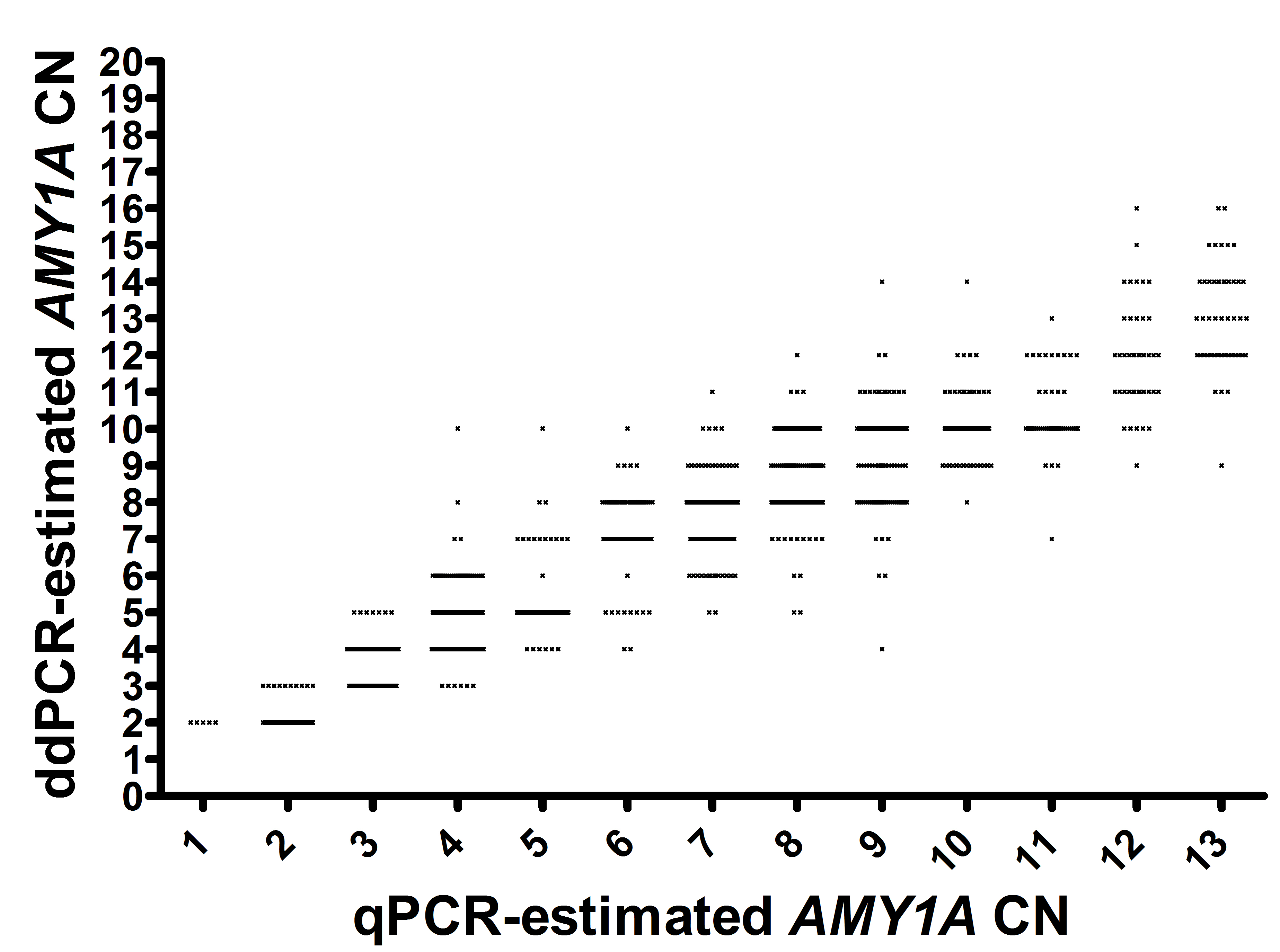


***CN*,** copy number
