# Supplementary material for: Relationship between salivary/pancreatic amylase and body mass index: a systems biology approach
Source: BMC Med. 2017 Feb 23;15:37. doi: 10.1186/s12916-017-0784-x (PMC5322607; doi:10.1186/s12916-017-0784-x)
Supplement: Additional file 9: — Association between ddPCR-estimated AMY1A copy number and BMI in several sets of D.E.S.I.R.: (i) the first set of 2137 samples previously analyzed by qPCR in Falchi et al. [3] paper; (ii) the second set of “all samples minus those 2137 samples”, and (iii) all samples from D.E.S.I.R. (DOC 31 kb) [file 12916_2017_784_MOESM9_ESM.doc]

**Additional file 9. Association between ddPCR-estimated *AMY1A*** copy number and BMI in several sets of D.E.S.I.R.: i/ the first set of 2,137 samples previously analyzed by qPCR in Falchi et al. paper; ii/ the second set of “all samples minus those 2,137 samples” and iii/ all samples from D.E.S.I.R

|  | ***n*** | ***AMY1A* copies*** | **Effect size ± SE**^†^  **per *AMY1A* copy** | ***p*-value** | **Effect size ± SE**^‡^  **per *AMY1A* copy** | ***p*-value** |
| --- | --- | --- | --- | --- | --- | --- |
| **“First set”** | 2,137 | 7.1 ± 2.7 | -0.0043 ± 0.0011 | 1.7 × 10^-4^ | -0.0044 ± 0.0011 | 1.5 × 10^-4^ |
| **“Second set”** | 1,463 | 6.7 ± 2.5 | -0.0006 ± 0.0012 | 0.62 | -0.0007 ± 0.0013 | 0.57 |
| **All samples** | 3,600 | 6.9 ± 2.6 | -0.0018 ± 0.0009 | 0.044 | -0.0018 ± 0.0009 | 0.041 |

*Data are means ± standard deviation

^†^Effect size according to linear regression model adjusted for age, sex, daily alcohol consumption, current smoking status, and the first two principal components for ethnicity;

^‡^Effect size according to linear regression model adjusted for age and sex.

BMI was logarithmically transformed before statistical analysis.
