# Supplementary material for: Relationship between salivary/pancreatic amylase and body mass index: a systems biology approach
Source: BMC Med. 2017 Feb 23;15:37. doi: 10.1186/s12916-017-0784-x (PMC5322607; doi:10.1186/s12916-017-0784-x)
Supplement: Additional file 10: — BMI (means ± s.e.m.) according to ddPCR-estimated AMY1A copy number in several sets of D.E.S.I.R.: (i) the first set of 2137 samples previously analyzed by qPCR in Falchi et al. [3] paper; (ii) the second set of “all samples minus those 2137 samples (=1463 samples)”, and (iii) all samples from D.E.S.I.R. (DOC 187 kb) [file 12916_2017_784_MOESM10_ESM.doc]

**Additional file 10. BMI (means ± s.e.m.) according to ddPCR-estimated *AMY1A* copy number in several sets of D.E.S.I.R.: i/ the first set of 2,137 samples previously analyzed by qPCR in Falchi et al. paper; ii/ the second set of “all samples minus those 2,137 samples (=1,463 samples)” and iii/ all samples from D.E.S.I.R**


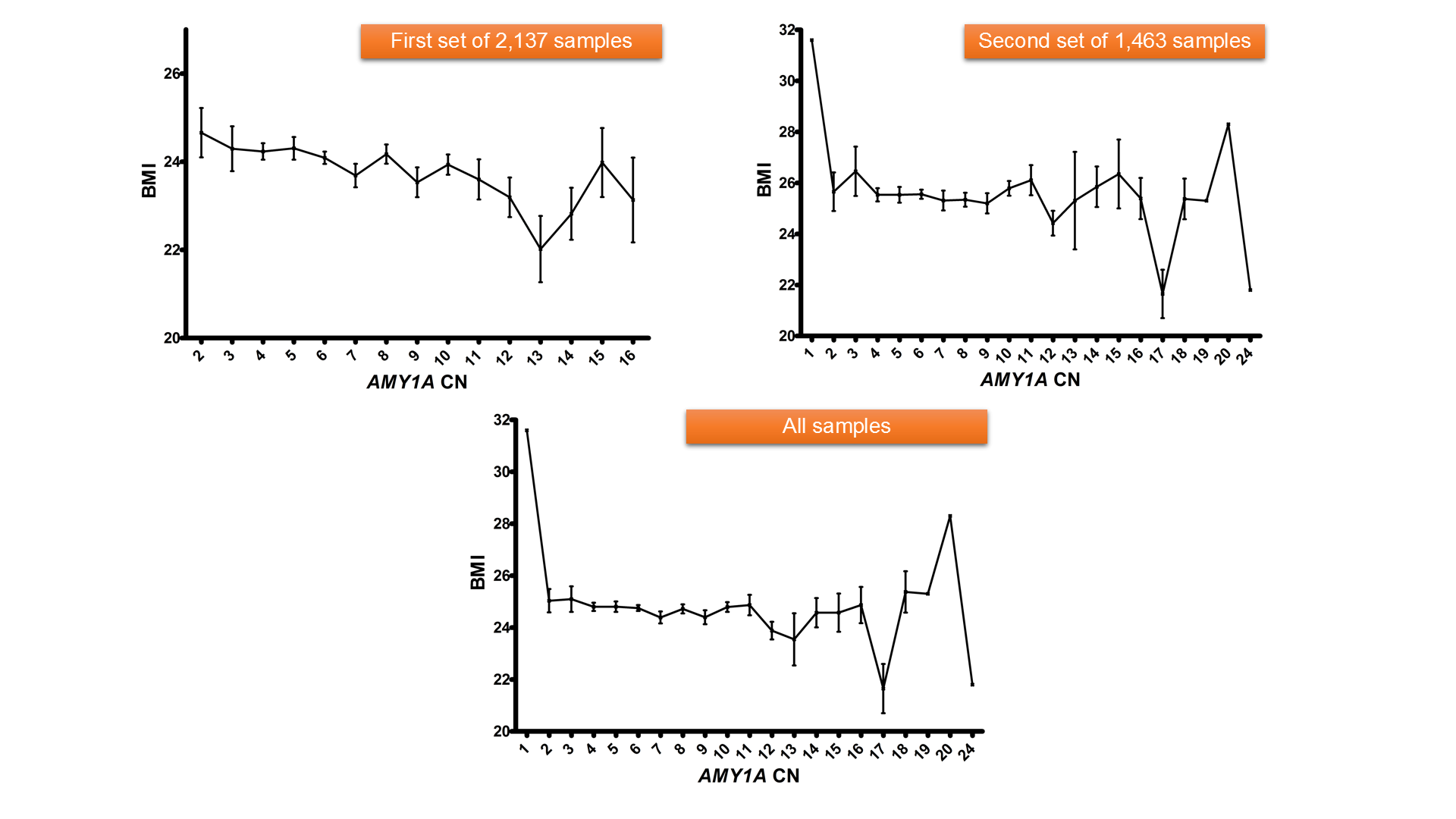


***CN*,** copy number.

All available data are reported.
