# Supplementary material for: Relationship between salivary/pancreatic amylase and body mass index: a systems biology approach
Source: BMC Med. 2017 Feb 23;15:37. doi: 10.1186/s12916-017-0784-x (PMC5322607; doi:10.1186/s12916-017-0784-x)

**Additional file 11. Distribution of *AMY1A* copy number in (A) 1,179 obese adults and 2,220 controls; (B) 785 obese children/adolescents and 712 controls**


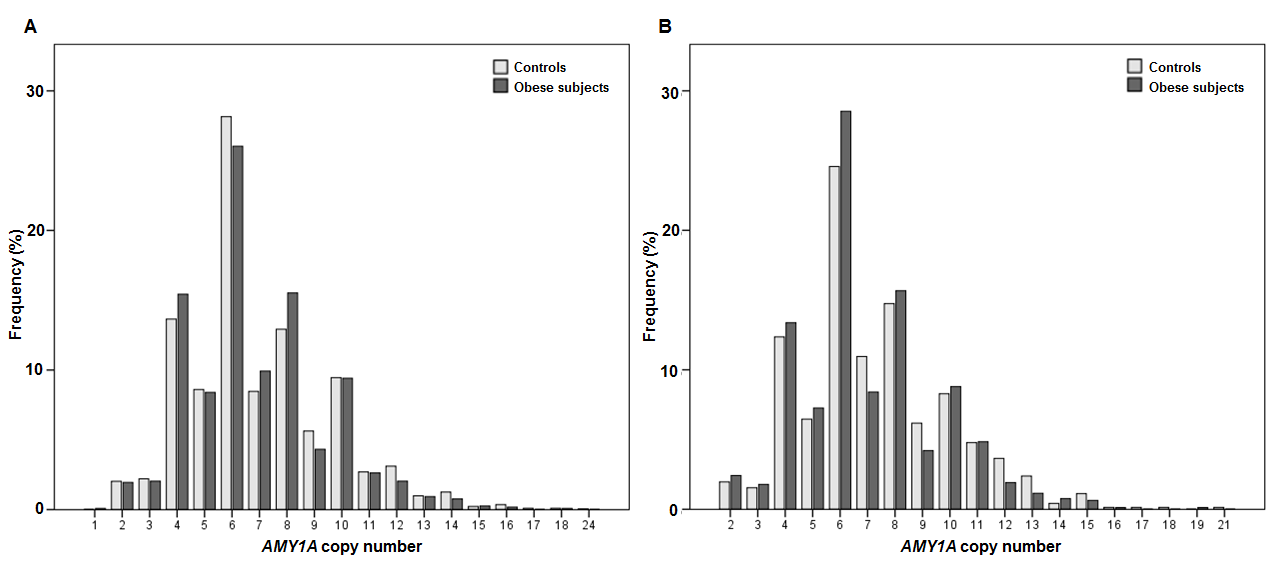

Supplement: Additional file 11: — Distribution of AMY1A copy number in (A) 1179 obese adults and 2220 controls; (B) 785 obese children/adolescents and 712 controls. (DOC 59 kb) [file 12916_2017_784_MOESM11_ESM.doc]
