# Supplementary material for: Relationship between salivary/pancreatic amylase and body mass index: a systems biology approach
Source: BMC Med. 2017 Feb 23;15:37. doi: 10.1186/s12916-017-0784-x (PMC5322607; doi:10.1186/s12916-017-0784-x)
Supplement: Additional file 13: — Significant associations between BMI-associated plasma metabolites and AMY1 or AMY2 plasma enzymatic activity in 718 participants from D.E.S.I.R. (DOC 47 kb) [file 12916_2017_784_MOESM13_ESM.doc]

**Additional file 13. Significant associations between BMI-associated plasma metabolites an**d AMY1 or AMY2 plasma enzymatic activity in 718 participants from D.E.S.I.R.

| **Metabolites** |  | **Sub-pathway** | **Effect size direction on BMI** | **AMY1 activity** | | | | **AMY2 activity** | | | |
| --- | --- | --- | --- | --- | --- | --- | --- | --- | --- | --- | --- |
|  | **Super-pathway** |  |  | **Without adjustment for BMI** | | **BMI-adjusted** | | **Without adjustment for BMI** | | **BMI-adjusted** | |
|  |  |  |  | **Effect size ± SE** | ***p*** | **Effect size ± SE** | ***p*** | **Effect size ± SE** | ***p*** | **Effect size ± SE** | ***p*** |
| 2-hydroxybuty-rate (AHB) | Amino Acid | Methionine, Cysteine, SAM and Taurine Metabolism | + | **-0.047±0.023** | **0.019** | -0.039±0.024 | 0.056 | -0.018±0.016 | 0.14 | -0.008±0.185 | 0.52 |
| Carnitine | Lipid | Carnitine Metabolism | + | 0.007±0.010 | 0.74 | 0.013±0.81 | 0.51 | **-0.027±0.013** | **0.021** | -0.020±0.015 | 0.087 |
| Glycerol | Lipid | Glycerolipid Metabolism | + | 0.011±0.043 | 0.60 | 0.023±0.037 | 0.26 | **-0.029±0.014** | **0.016** | -0.017±0.017 | 0.15 |
| Histidine | Amino Acid | Histidine Metabolism | - | -0.025±0.028 | 0.18 | -0.032±0.024 | 0.091 | **0.029±0.012** | **8.5×10^-3^** | **0.023±0.013** | **0.037** |
| Isoleucine | Amino Acid | Leucine, Isoleucine and Valine Metabolism | + | **-0.044±0.025** | **0.041** | -0.032±0.032 | 0.16 | 0.012±0.031 | 0.35 | 0.032±0.015 | 0.14 |
| Isovaleryl-carnitine | Amino Acid | Leucine, Isoleucine and Valine Metabolism | + | **-0.044±0.025** | **0.040** | -0.034±0.029 | 0.12 | -0.012±0.032 | 0.36 | 0.003±0.004 | 0.79 |
| Lactate | Carbo-hydrate | Glycolysis, Gluconeogenesis, and Pyruvate Metabolism | + | **0.050±0.020** | **5.8×10^-3^** | **0.058±0.020** | **1.6×10^-3^** | 0.0004±0.0002 | 0.97 | 0.008±0.050 | 0.43 |
| Leucine | Amino Acid | Leucine, Isoleucine and Valine Metabolism | + | **-0.062±0.025** | **6.5×10^-3^** | **-0.050±0.028** | **0.037** | -0.006±0.012 | 0.68 | 0.017±0.023 | 0.23 |
| N-acetylglycine | Amino Acid | Glycine, Serine and Threonine Metabolism | - | -0.001±0.001 | 0.94 | -0.014±0.30 | 0.48 | **0.023±0.013** | **0.036** | 0.011±0.026 | 0.34 |
| Phenylalanine | Amino Acid | Phenylalanine and Tyrosine Metabolism | + | **-0.040±0.023** | **0.042** | -0.029±0.029 | 0.15 | -0.020±0.014 | 0.084 | -0.004±0.008 | 0.72 |
| Tyrosine | Amino Acid | Phenylalanine and Tyrosine Metabolism | + | -0.030±0.025 | 0.11 | -0.017±0.060 | 0.39 | **-0.030±0.013** | **8.1×10^-3^** | -0.015±0.019 | 0.21 |

***AMY1***, salivary amylase; ***AMY2***, pancreatic amylase; ***BMI***, body mass index; ***SE***, standard error.
